# Congenital prepubic sinus with dorsal penile curvature: a case report and literature review

**DOI:** 10.1186/s12887-019-1768-0

**Published:** 2019-10-22

**Authors:** Chuan Wang, Xue Ma

**Affiliations:** Department of Pediatric Surgery, West China Hospital of Sichuan University, #37 Guo-Xue-Xiang, Chengdu, 610041 Sichuan Province China

**Keywords:** Congenital prepubic sinus, Dorsal penile curvature, Urethral duplication

## Abstract

**Background:**

Congenital prepubic sinus (CPS) is a rare congenital anomaly and widely thought to be a variant of urethral duplication. Histological examination of this case gives a clue to this theory. CPS with dorsal penile curvature has been reported in previous publications, but their procedures to correct the curvature are different from this case.

**Case presentation:**

A 10-year-old boy complained of the pain in the dorsal base of the penis. Physical examination revealed an accessory meatus located in the midline of the dorsal proximal penis and moderate dorsal penile curvature with deficient dorsal foreskin. Imaging examination showed that the meatus did not communicate with either normal urethra or urinary bladder, and ended blindly at the level of the symphysis pubis. The intact 4-cm-long sinus was completely separated and excised. Penile curvature was corrected after the dorsal proximal fibrous cord was detached. Histological examination confirmed the diagnosis of urethral duplication.

**Conclusions:**

The histological result of this case supports the theory that CPS is a variant of the dorsal urethra. Moreover, this case indicates that the curvature in patients with CPS may be caused by the dorsal fibrous cord at the beginning and the operation should be conducted at an early age to avoid further development of the curvature during puberty.

## Background

Urethral duplication is an infrequent congenital lower urinary tract anomaly and generally classified into three types, including incomplete (type 1), complete (type 2) and coronal (type 3) [1–3]. Congenital prepubic sinus (CPS) is suggested as a variant of incomplete dorsal urethral duplication by immunohistochemical analysis [4]. CPS represents a blind-ending duplicated urethra located in front of the pubic symphysis, which stretches to, but not communicates with urinary bladder. Patients often complain of intermittent mucous or purulent discharge from the meatus. Here, we present an interesting case of CPS companied by dorsal penile curvature, with the chief complaint of the pain in the dorsal base of the penis.

## Case presentation

**Fig. 1:**
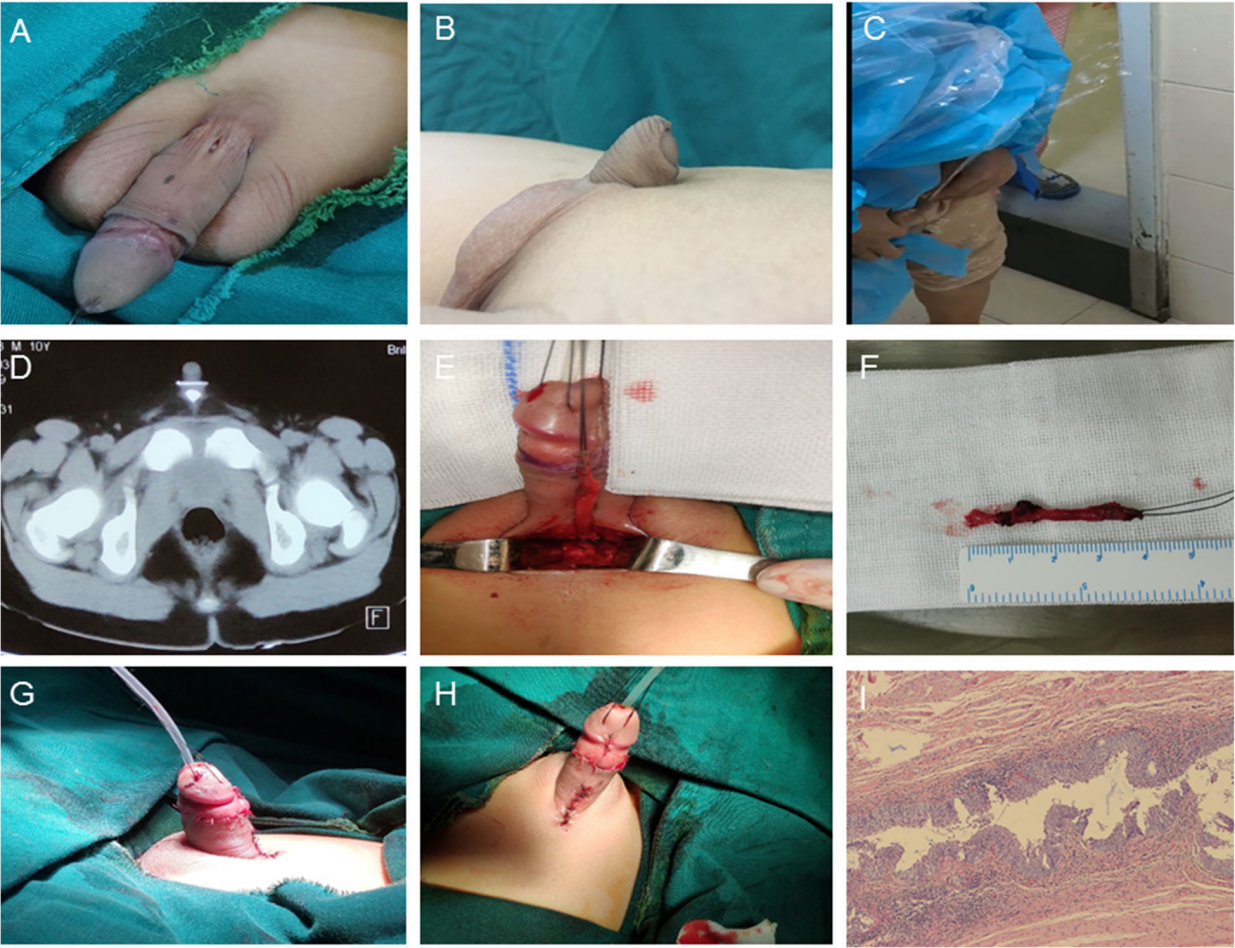
**a** Preoperative photograph showing an accessory meatus located in the midline of the dorsal proximal penis. **b** Preoperative photograph showing dorsal penile curvature with deficient dorsal foreskin. **c** Preoperative photograph showing the abnormal direction of the urinary stream. **d** CT with contrast medium in sinus showing bind ending not communicating with either normal urethra or urinary bladder. **e** and **f** Intraoperative photograph showing the complete sinus. **g** and **h** Postoperative photograph presenting the penis after correction. **i** Histological examination showing transitional epithelium lining inside the sinus and a few smooth muscle bundles around the sinus

A 10-year-old Chinese boy was admitted to our hospital with the chief complaint of the pain in the dorsal base of the penis. He presented with occasional discharge from an accessory meatus located in the midline of the dorsal proximal penis, combined with dorsal penile curvature (Fig. 1a, b). The curvature made the urine easily spray to his face when he urinated without pushing the penis down (Fig. 1c). Seven months ago, he suffered from the pain in the dorsal base of the penis and the skin around the abnormal opening was red and swollen when the meatus soaked in sweat after vigorous activities.

Physical examination revealed normally developing bilateral testicles and penis, and urethral meatus at the normal position of the glans. There was an accessory meatus located in the midline of the dorsal proximal penis. Moderate dorsal penile curvature was identified with deficient dorsal foreskin. No remarkable abnormality was found in hematological, biochemical, urinary, or endocrinologic tests. Patent foramen ovale, which required no intervention, was identified by echocardiography. Retrograde urethrogram, retrograde sinogram and computed tomography (CT) showed that the epispadiac tract did not communicate with either normal urethra or urinary bladder, and ended blindly at the level of the symphysis pubis (Fig. 1d). Thus, CPS was diagnosed.

Methylthionine chloride was injected into the sinus through the dorsal opening at the initiation of the procedure, and the intact 4-cm-long sinus was completely separated and excised (Fig. 1e, f). Then a circumcoronal incision was made and the skin coverings of the shaft of the penis were taken down. Penile curvature was corrected after dorsal proximal fibrous cord beneath the skin was detached (Fig. 1g, h). The patient was discharged from hospital uneventfully.

Histological examination showed that the lining epithelium inside sinus was transitional epithelium, and a few smooth muscle bundles were identified around the sinus (Fig. 1i). These findings confirmed the diagnosis of urethral duplication.

## Discussion and conclusions

CPS is an extremely rare congenital anomaly. Two theories have been proposed to illustrate its etiology, including a variant of dorsal urethral duplication and an abdominal wall closure defect. In this case, the histological result supports the theory that CPS is a variant of the dorsal urethra duplication, which is consistent with other studies [4, 5]. CPS is distinct from ventral urethra duplication. Ventral urethra duplication usually communicates with lower urinary tract and rarely companied with penile curvature.

CPS with dorsal penile curvature has been reported in previous publications [6, 7]. Two patients were 14 and 20 years old, respectively, and presented with intermittent discharge from opening of CPS as our case presented. Dorsal penile curvatures in both cases were corrected, but the procedures were different from our case. The curvature was corrected by conducting two pairs of ventral corporal plication in the previous two cases. However, the curvature was easily corrected by detaching the dorsal proximal fibrous cord in our case. The different procedures in these cases indicate that the curvature in patients with CPS may be caused by the dorsal fibrous cord at the beginning and the operation should be conducted at an early age to avoid further development of the corpus cavernosum curvature during puberty.

Retrograde urethrogram, retrograde sinogram, and CT were all conducted in this case. However, CT seems not to provide additional information compared to retrograde urethrogram plus retrograde sinogram in terms of the relationship between CPS and lower urinary tract. Thus, we suggest performing ultrasound rather than CT to investigate the upper urinary tract, which makes much less radiation exposure to the patients.

## Data Availability

The datasets used during the current study are available from the corresponding author on reasonable request.

## Abbreviations

CPS: Congenital prepubic sinus
CT: Computed tomography
